# Transcriptional analysis of microRNAs related to unsaturated fatty acid synthesis by interfering bovine adipocyte ACSL1 gene

**DOI:** 10.3389/fgene.2022.994806

**Published:** 2022-09-26

**Authors:** Xupeng Li, Yanbin Bai, Jingsheng Li, Zongchang Chen, Yong Ma, Bingang Shi, Xiangmin Han, Yuzhu Luo, Jiang Hu, Jiqing Wang, Xiu Liu, Shaobin Li, Zhidong Zhao

**Affiliations:** Gansu Key Laboratory of Herbivorous Animal Biotechnology, College of Animal Science and Technology, Gansu Agricultural University, Lanzhou, China

**Keywords:** micrornas, ACSL1, bovine adipocytes, unsaturated fatty acids, RNA-seq

## Abstract

Long-chain fatty acyl-CoA synthase 1 (ACSL1) plays a vital role in the synthesis and metabolism of fatty acids. The proportion of highly unsaturated fatty acids in beef not only affects the flavor and improves the meat’s nutritional value. In this study, si-ACSL1 and NC-ACSL1 were transfected in bovine preadipocytes, respectively, collected cells were isolated on the fourth day of induction, and then RNA-Seq technology was used to screen miRNAs related to unsaturated fatty acid synthesis. A total of 1,075 miRNAs were characterized as differentially expressed miRNAs (DE-miRNAs), of which the expressions of 16 miRNAs were upregulated, and that of 12 were downregulated. Gene ontology analysis indicated that the target genes of DE-miRNAs were mainly involved in biological regulation and metabolic processes. Additionally, KEGG (Kyoto Encyclopedia of Genes and Genomes) pathway analysis identified that the target genes of DE-miRNAs were mainly enriched in metabolic pathways, fatty acid metabolism, PI3K-Akt signaling pathway, glycerophospholipid metabolism, fatty acid elongation, and glucagon signaling pathway. Combined with the previous mRNA sequencing results, several key miRNA-mRNA targeting relationship pairs, i.e., novel-m0035-5p—ACSL1, novel-m0035-5p—ELOVL4, miR-9-X—ACSL1, bta-miR-677—ACSL1, miR-129-X—ELOVL4, and bta-miR-485—FADS2 were screened via the miRNA-mRNA interaction network. Thus, the results of this study provide a theoretical basis for further research on miRNA regulation of unsaturated fatty acid synthesis in bovine adipocytes.

## Introduction

Fat deposits and unsaturated fatty acid (UFA) content in beef muscle not only affect meat quality and flavor but also help improve the nutritional value of meat. Polyunsaturated fatty acids (PUFAs) play a significant role in chronic diseases, including cardiovascular disorders, cancers, and diabetes mellitus (Sijben and Calder, 2007; Marventano et al., 2015). Such as, long-chain n−3 polyunsaturated fatty acids, eicosapentaenoic acid (EPA, 20:5n−3) and docosahexaenoic acid (DHA; 22:6n−3) can reduce cardiovascular disease, the risks of cancer and type 2 diabetes have been widely recognized (Simopoulos, 1991; Barcelo-Coblijn and Murphy, 2009; Lopez-Huertas, 2010). Long-chain fatty acyl-CoA synthetases (ACSLs) are essential for fatty acid (FA) activation and catalysis. *ACSL1* converts long-chain FAs to fatty acyl-CoA (Paul et al., 2014). Previous studies have shown that *ACSL1* plays an important role in the activation of fatty acid synthesis of triglyceride (Li et al., 2009). In addition, *ACSL1* gene variants are also known to affect the content of unsaturated omega-3 FA, PUFAs, long-chain omega-3 FA, and docosapentaenoic acid in bovine skeletal muscle (Widmann et al., 2011). The results of follow-up studies have also shown that interference with the *ACSL1* gene caused reduced levels of MUFAs and PUFAs in bovine adipocytes. In contrast, the overexpression of the *ACSL1* gene was found to significantly upregulate the content of PUFAs (Tian et al., 2020; Zhao et al., 2020). Related studies have also found that miRNAs regulate lipid metabolism in milk (Lian et al., 2016), liver cancer cells (Cui et al., 2014), laying hens (Tian et al., 2019), and pig subcutaneous fat (Shan et al., 2022) by targeting *ACSL1*. Based on these results, *ACSL1* plays a key regulatory role in the synthesis of unsaturated fatty acids.

MicroRNAs (miRNAs) are endogenous non-coding small RNAs (approximately 22 nt long), which are widely found in eukaryotic cells (Shukla et al., 2011). They bind to the specific complementary site of the target mRNA at the post-transcriptional level, thereby degrading the mRNA or inhibiting its translation and regulating the protein expression (Bartel, 2009). Previous studies found that miRNAs play an important regulatory role in cell proliferation and differentiation, early animal development, apoptosis, oncogene expression inhibition, and fat metabolism (Tufekci et al., 2014; Deb et al., 2018). Many miRNAs are known to regulate the differentiation and deposition of adipocytes by regulating *PPAR*, C/EBPs, and other transcription factor families (Shukla et al., 2011). For example, bta-miR-130a/b is known to affect the differentiation of bovine adipocytes by targeting *PPARG* and *CYP2U1* (Ma et al., 2018). Also, miR-27a inhibits the differentiation of sheep preadipocytes by specifically binding to *RXRα* 3′UTR (Deng et al., 2020). Han et al. also found that miR-193a-5p inhibited the proliferation and differentiation of sheep preadipocytes by targeting the *ACAA2* gene (Han et al., 2021). A recent study found that miRNAs were involved in the cascade of genes and transcription factors to regulate fatty acid desaturation and fat deposition (Ren et al., 2020). These studies indicated that miRNAs exhibited a critical regulatory effect on fat metabolism.

This study aimed to further explore the molecular mechanism of miRNAs regulating the synthesis of unsaturated fatty acids in bovine adipocytes. High-throughput sequencing was used to systematically identify miRNAs and mRNAs (Bai et al., 2021) that were differentially expressed in bovine adipocytes after interference with the *ACSL1* gene and linked the mRNAs related to fatty acid metabolism with miRNAs to construct a miRNA-mRNA interaction network. The results provide new ideas and directions for the regulation of unsaturated fatty acid metabolism in bovine adipocytes.

## Materials and methods

### Sample collection

This animal study was reviewed and approved by the Faculty Animal Policy and Welfare Committee of Gansu Agricultural University (Ethic approval file No. GSAU-Eth-AST-2021-25). Calves (aged 1 day) from the livestock ranch of Gansu Agricultural University (Lanzhou, China) were selected. After sacrificing the calf, the perirenal adipose tissue was immediately collected for the isolation of bovine preadipocytes. The specific method of cell isolation is the same as our previous research (Tian et al., 2020).

### Bovine pre-adipocyte culture and cell transfection

The bovine preadipocytes were cultured to the F3 generation, and then differentiation was induced *in vitro*. The test operation process and method followed the methods used in our previous studies (Tian et al., 2020). Finally, the cells on the fourth day of differentiation were collected in a 1.5 ml centrifuge tube without ribonuclease and were rapidly frozen in liquid nitrogen for subsequent RNA sequence analysis.

### Construction and sequencing of small ribonucleic acid library

Total RNA was extracted from six adipocyte samples (3 with *ACSL1* gene interference, si group; the other three without *ACSL1* gene interference, NC group) using the Trizol kit (Invitrogen, Carlsbad, CA, USA). RNA degradation and contamination were analyzed via 1% agarose gel electrophoresis; RNA purity (OD260/280 ratio) was determined by Nanodrop analysis; RNA concentration was accurately quantified via Qubit; RNA integrity was determined using Agilent 2,100. After quantification, the NEBNext^®^ Multiplex Small RNA Library Prep Set for Illumina^®^ (NEB, USA) was used to build the library. Using total RNA as the starting sample, polyacrylamide gel (PAGE) electrophoresis was run, select bands in the range of 18–30 nt were isolated, and recovers small RNA. Connect the 3′ adaptor and the 5’ adaptor, respectively, and then perform reverse transcription and PCR amplification on the small RNAs connected to the adaptors on both sides. Finally, the PAGE gel was used to recover and purify the 140 bp band and dissolve it in EB solution to complete the library construction. The constructed library was tested for quality and yield using Agilent 2,100 and ABI StepOnePlus Real-Time PCR System (Life Technologies). After passing the quality inspection, the sequencing was performed by Novogene Bioinformatics Institute (Beijing, China) on Illumina HiSeq 2,500.

### Identification of differential miRNAs

Raw reads were further filtered according to the following rules: 1) low quality reads containing more than one low quality (Q-value≤20) base or containing unknown nucleotides (N) were removed; 2) reads without 3′ adapters were removed; 3) reads containing 5′ adapters were removed; 4) reads containing 3′ and 5’ adapters but no small RNA fragment between them were removed; 5) reads containing ployA in small RNA fragment were removed. After the quality control process, the clean tag sequence was aligned to the reference genome of the known cattle (Bos_taurus_Ensembl_94), and classification was performed by aligning it with the GeneBank (version 209.0), Rfam (11.0), and miRBase (22.0) databases. The sequence matching miRBase was considered as the known miRNA. According to software mirdeep2, new miRNA candidates were identified. The miRNA expression level was calculated and normalized to transcripts per million (TPM). For differentially expressed miRNA analysis, DESeq2 (V1.20.0) software was used. The screening criteria for differential miRNAs were the | fold change | > 2.0 and *p*-value < 0.05. The differential expression of all miRNAs, existing miRNAs, known miRNAs, and new miRNAs were analyzed simultaneously.

### Functional enrichment analysis of differential miRNAs

Miranda (v3.3a) and TargetScan (Version: 7.0), two prediction software, were used to predict the target gene of miRNA, and the intersection of the target gene prediction results was used as the result of miRNA target gene prediction. The miRNA target genes were mapped to each term of the GO database (http://www.geneontology.org/), and the number of miRNA target genes was calculated for each term to obtain a list of miRNA target genes with a GO function miRNA target statistics of the number of genes. The hypergeometric test was used to find GO entries that were significantly enriched in miRNA target genes compared with the background. KEGG (Kyoto Encyclopedia of Genes and Genomes) enrichment analysis used hypergeometric testing to find pathways that were significantly enriched in miRNA target genes compared with the entire background. Pathway significant enrichment was used to determine the most important biochemical metabolic pathways and signal transduction pathways involved in miRNA target genes. Finally, GO and KEGG pathways with Q-value < 0.05 were selected as the ones that were significantly enriched.

### Real-time PCR verification

Ten miRNAs were selected for qRT-PCR to verify the differential expression results of sequencing. For miRNA expression, a miRNA first-strand cDNA synthesis kit (Accurate Biology, Hunan, China) was used to perform real-time fluorescent quantitative PCR. U6 snRNA was used as an internal reference. All qRT-PCR reactions were performed in the ABI 7500 real-time PCR system (Applied Biosystems, California, USA), with three reactions/samples. The relative expression of miRNA was calculated using the 2^−ΔΔCt^ method (Livak and Schmittgen, 2001).

### Construction of miRNA-mRNA interaction network

We selected nine signaling pathways for miRNA target gene enrichment, which are related to lipid metabolism. Then we intersect the genes enriched in the signaling path with the mRNAs expressed differently in our previous sequencing results (Bai et al., 2021), and use these mRNAs and miRNAs to build a miRNA-mRNA interaction network.

## Results

### Overview of small RNA sequencing

**FIGURE 1:**
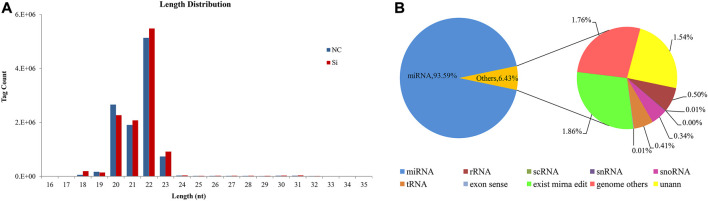
Correlation analysis of small RNA sequencing data. **(A)** Statistical analysis of small RNA fragment size. **(B)** Statistical analysis of the types of small RNA fragments after comparison with the database, including miRNA (existing miRNAs, known miRNAs and novel miRNAs), rRNA, scRNA, snRNA, snoRNA, tRNA, exon sense, miRNA editing, other genome, and unann.

After high-throughput sequencing of six bovine adipocytes, the average clean reads of NC and si were 11, 368, 893 and 11,869,071, respectively, and the average clean tags obtained after quality control were 10,845,625 (95.40%) and 11,302,810 (95.23%) (Table 1). The raw reads obtained in the study were submitted to GenBank Temporary Submission ID: SUB11745359. The length of the smallest RNAs was in the range of 18–23 nt. The miRNAs of 22 nt were the longest, followed by miRNAs with a length of 20 nt, 21 nt, and 23 nt (Figure 1A). After comparing with GenBank, Rfam, and the reference genome, 93.59% of clean reads were identified as miRNA, and the remaining part included rRNA, scRNA, snRNA, snoRNA, tRNA, miRNA editing, and unann (Figure 1B). Approximately 96.15% of the tags were aligned to the reference genome (Bos_taurus_Ensembl_94). There were 491 bovine miRNAs and 462 known miRNAs. In addition, 122 new miRNAs were also predicted (Supplementary File S1).

**TABLE 1:** Overview of small RNA sequencing.

Id	clean_reads	high_quality	3’adapter_null	insert_null	5’adapter_contaminants	polyA	clean_tags
NC-1	11,655,387 (100%)	11,568,102 (99.2511%)	26,054 (0.2252%)	49,674 (0.4294%)	14,982 (0.1295%)	103 (0.0009%)	10,986,690 (94.2628%)
NC-2	11,231,395 (100%)	11,097,944 (98.8118%)	59,021 (0.5318%)	4,456 (0.0402%)	3,871 (0.0349%)	112 (0.0010%)	10,857,341 (96.6696%)
NC-3	11,219,898 (100%)	11,079,983 (98.7530%)	33,458 (0.3020%)	8,190 (0.0739%)	7,796 (0.0704%)	110 (0.0010%)	10,692,844 (95.3025%)
si-1	11,463,101 (100%)	11,357,344 (99.0774%)	25,051 (0.2206%)	96,814 (0.8524%)	21,465 (0.1890%)	145 (0.0013%)	1,0,491,794 (91.5267%)
si-2	12,463,501 (100%)	1,2,301,997 (98.7042%)	48,443 (0.3938%)	6,041 (0.0491%)	4,318 (0.0351%)	125 (0.0010%)	12,072,144 (96.8600%)
si-3	11,680,612 (100%)	11,550,211 (98.8836%)	58,475 (0.5063%)	4,189 (0.0363%)	3,364 (0.0291%)	84 (0.0007%)	11,344,492 (97.1224%)

### Analysis of differentially expressed miRNAs

**FIGURE 2:**
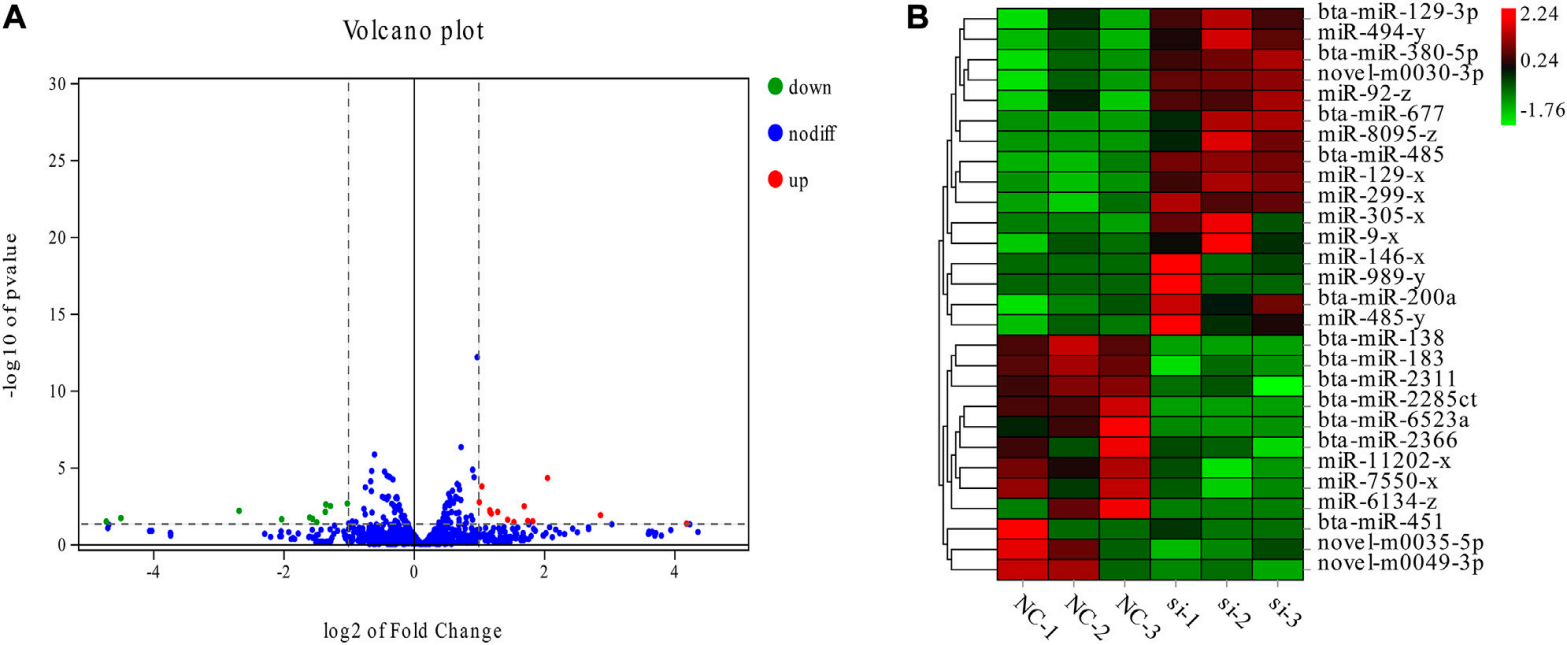
Statistical analysis of DE-miRNA. **(A)** The expression of miRNA volcano. The green dots on the left represent miRNAs that are significantly downregulated; the blue dots represent miRNAs that were not significantly different; and the red dots on the right represent miRNAs that were significantly upregulated. **(B)** Cluster map of differentially expressed miRNAs. Red indicates elevated expression and green indicates downregulated expression.

The DESeq2 (V1.20.0) software was used to compare the miRNAs between the si group and the NC group, and 28 DE-miRNAs were obtained, including 25 known and three new miRNAs. Among them, 16 upregulated miRNAs and 12 downregulated miRNAs were in the si group (Figure 2A). In general, the difference between the interference *ACSL1* gene (si group) and the control group (NC group) was highly correlated with the expression of these miRNAs. The clustering patterns of these 28 DE-miRNAs are shown in (Figure 2B).

### GO and KEGG enrichment analysis of differentially expressed miRNA

**FIGURE 3:**
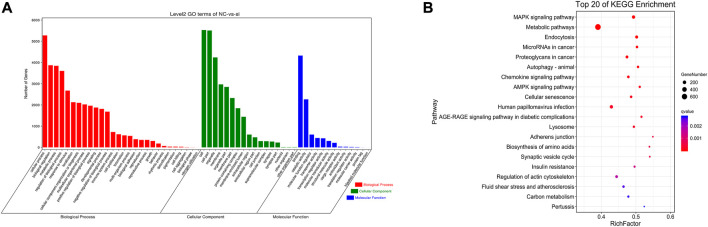
Functional enrichment analysis of DE-miRNAs. **(A)** GO enrichment analysis of target genes of DE-miRNAs. **(B)** Top 20 KEGG signaling pathways enriched by DE-miRNAs target genes.

TargetScan (Version: 7.0) and miRanda (v3.3a) was used to predict DE-miRNA target genes to determine their biological functions. The 28 DE-miRNAs predicted a total of 6,828 target genes (Supplementary File S2). GO enrichment analysis showed that these target genes participated in 1,111 significantly enriched functional classifications (Q-value < 0.05). Biological processes contained the most enriched genes with 817 GO terms, followed by cell components with 203 GO terms and molecular functions with 91 GO terms (Supplementary File S3). The enriched GO terms were mainly related to cellular processes, biological regulation, metabolic processes, cellular parts, binding, and catalytic activity (Figure 3A). KEGG results indicated that the target genes of DE-miRNAs were significantly enriched in 81 signaling pathways (Q-value < 0.05) (Supplementary File S4). The enriched pathways included MAPK signaling pathway, metabolic pathway, AMPK signaling pathway, fatty acid metabolism, PI3K-Akt signaling pathway, glycerophospholipid metabolism, fatty acid elongation, steroid biosynthesis, and glucagon signaling pathway (Figure 3B).

### Validation of differentially expressed miRNAs by qRT-PCR

**FIGURE 4:**
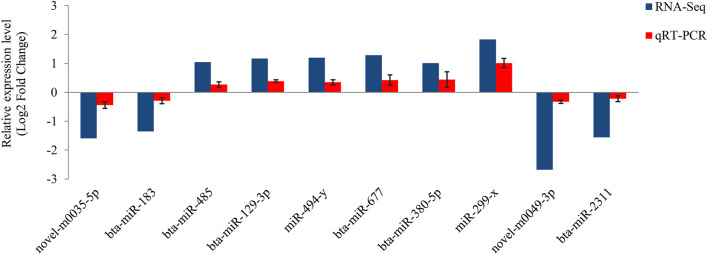
Validation of the miRNA-Seq results. The qRT-PCR validation was performed on 10 DE-miRNAs. These data show the mean ± SD for three replicates. Error bars indicate the standard deviation.

The relative expression of 10 miRNAs was quantified by qRT-PCR to verify the differentially expressed miRNA (Figure 4). All selected DE-miRNAs showed consistent expression patterns between RNA-seq and qRT-PCR results, confirming the sequencing results.

### Construction of the miRNA-mRNA interaction network

**FIGURE 5:**
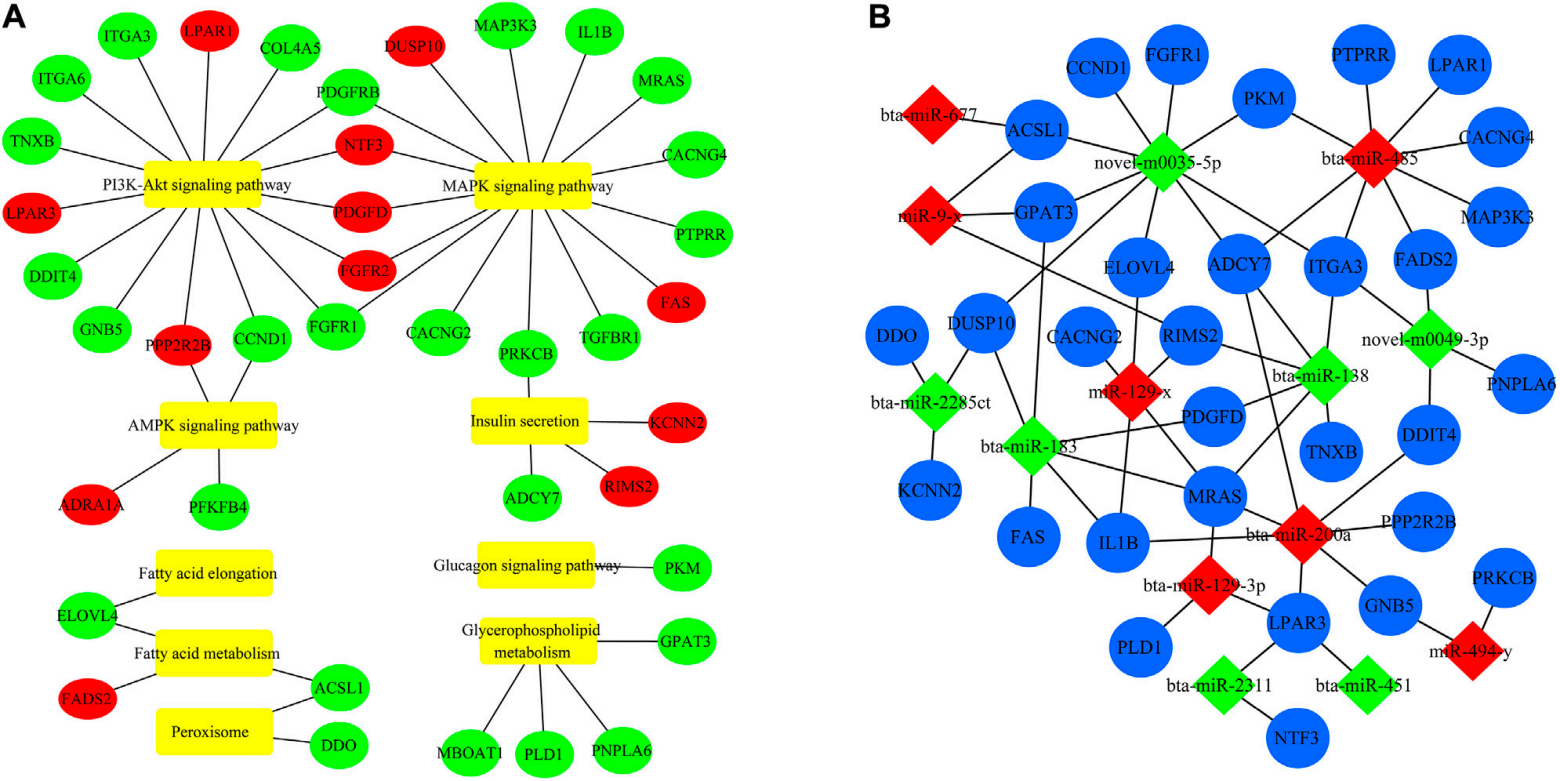
Interaction network of DE-miRNAs. **(A)** Network analysis of DE-miRNA target genes and their enrichment pathways. Red shows the upregulated mRNA. Green represents downregulated mRNA, and yellow represents the enriched signaling pathways. **(B)** Co-expression network of DE-miRNA and its target genes. Red is the miRNA whose expression is upregulated. Green represents downregulated miRNA, and blue represents mRNA.

The background genes related to fatty acid metabolism signaling pathways were screened to further understand and visualize the interaction between fat-related differentially expressed mRNAs (DEMs) and differentially expressed miRNAs (DE-miRNAs) (Figure 5A). Then these genes were used as target genes to construct a miRNA-mRNA interaction network (Figure 5B). In the network, 60 interactions were identified, and several of the most important DE-miRNAs were novel-m0035-5p, bta-miR-485, bta-miR-200a, bta-miR-677, miR-9-x, and miR-129-x.

## Discussion

Fat deposition in muscle involves a series of processes, such as preadipocyte proliferation, preadipocyte differentiation, and lipid metabolism, and is regulated by related functional genes (Cai et al., 2018). MicroRNAs, as an important regulatory element after gene transcription, have been widely reported for their role in regulating lipid metabolism and synthesis (Du J et al., 2018; Jin et al., 2019; Khan et al., 2020; Zhang et al., 2020; Zhou et al., 2020). Therefore, it is necessary to understand the molecular mechanism of miRNAs in regulating the synthesis of unsaturated fatty acids (UFAs)-related genes to improve the nutritional value of beef.

In this study, 1,075 miRNAs were identified, of which 122 miRNAs were novel. Compared with the NC group, 28 miRNAs were identified as differentially expressed miRNAs. The DE-miRNAs included known lipid metabolism-related miRNAs such as miR-200a (Zhang et al., 2018), as well as newly discovered miRNAs novel-m0035-5p and novel-m0049-3p. GO and KEGG pathway enrichment analyses of miRNA target genes were carried out to reveal the potential function of DE-miRNAs. GO enrichment results showed that DE-miRNAs were mainly involved in cell processes, biological regulation, metabolic process, cellular parts, and binding. The results of KEGG analysis showed that DE-miRNAs target genes were mainly significantly enriched in 81 signal pathways (Q < 0.05). Nine pathways (Table 2) related to the synthesis of UFAs were selected for the follow-up research. The PI3K-Akt signaling pathway is a signaling pathway related to insulin (Pessin and Saltiel, 2000). Insulin regulates lipogenesis, fatty acid oxidation, VLDL-TG assembly, and secretion in goose liver cells through PI3K-Akt-mTOR to mediate lipid deposition (Han et al., 2015). Also, insulin and other hormones, such as glucagon, coordinate and regulate multiple biological responses. Insulin stimulates glucose transport in surrounding tissues, such as muscle and adipose tissue, inhibits glycogen synthesis and gluconeogenesis in the liver, and stimulates protein synthesis and lipogenesis (Wang and Sul, 1998). The MAPK signaling pathway can control biological processes via various cellular mechanisms involving the activation/inhibition of related factors (Yue and Lopez, 2020). Studies have found that miR-145 inhibits the lipid production of bovine preadipocytes by reducing the activity of PI3K/Akt and MAPK signaling pathways (Wang et al., 2020). Wu et al. found that miR-29a targeting *CTRP6* inhibited the proliferation of pig muscle and subcutaneous adipocytes via the MAPK signaling pathway but promoteed differentiation (Wu et al., 2017). AMPK is one of the important enzymes that regulate cell energy metabolism and participates in cell metabolism. Activating AMPK in 3T3-L1 cells can inhibit the expression of key genes *C/EBPβ*, *PPARγ*, and *C/EBPα* in adipogenesis (Lee et al., 2012). We also found that more genes related to fat differentiation, such as *PPARγ*, *FASN*, and *SCD5*, are enriched in the AMPK signaling pathway. The enzymes required for β-oxidation of fatty acyl-CoA are present in peroxisomes and mitochondria. These enzymes promote the β-oxidation of long-chain and ultra-long-chain fatty acids in peroxisomes (Latruffe et al., 2001). They may also play a key role in regulating cellular senescence in related biochemical processes (Titorenko and Terlecky, 2011). An miRNA-mRNA interaction network was constructed to screen out miRNAs and mRNAs that may be related to UFA synthesis more intuitively and accurately. Here nine signaling pathways related to lipid metabolism were selected and then the background genes in the pathways were intersected with the differential mRNA sequenced from the previous transcriptome (Bai et al., 2021). These intersecting mRNAs and differential miRNAs were used to construct an interaction network. In the interaction network, the novel-m0035-5p and bta-miR-485 were found to simultaneously target multiple differential mRNAs. Most of the research on miR-485 is related to human cancer. A recent report discovered that miR-485 affected the content of triglycerides (TGs) and cholesterol (CHOL) in milk fat by targeting the *DTX4* gene (Liu et al., 2021).

**TABLE 2:** Nine significantly enriched pathways related to lipid metabolism.

Pathyway ID	Pathyway term	Qvalue	Target gene list
ko04151	PI3K-Akt signaling pathway	2.39E-02	ANGPT2,COL1A2,COL9A1,FGFR1,PRKAA1,CDKN1A,FGF2,PRLR,COL4A1,COL4A5,LPAR1,CD19,MAPK3
			COL6A1,GNB3,LPAR5,FGFR2,G6PC,ITGA6,COL6A5,LPAR2,PPP2R1A,PPP2R2C,CREB1,THBS1
ko04010	MAPK signaling pathway	3.07E-08	ANGPT2,FGF18,MAP3K1,DUSP2,FGFR1,IKBKB, TGFB2,TEK,MAPK14,PPP3CB,DUSP10,FGF2,NF1,AKT3
			NLK,SRF,MAPK8,EFNA4,CACNB3,MET,RRAS2,TNF,IL1A,MAP3K4,AKT1,FAS
ko04152	AMPK signaling pathway	1.89E-04	PPP2CB,EIF4EBP1,SIRT1,PFKFB2,PRKAA1,CD36,AKT3,MAP3K7,CREB5,ADRA1A,SREBF1,ADIPOR2
			PPARGC1A,PPP2R1A,FASN,SCD5,CREB1,CCND1,HMGCR
ko00062	Fatty acid elongation	4.12E-02	ELOVL4,HACD4,ELOVL5,PPT2,HACD2,ACOT7,HSD17B12,HADHB, HADHA,ECHS1,ELOVL6,ELOVL1,HADH
			PPT1,HACD3,ACOT4
ko01212	Fatty acid metabolism	5.31E-03	ACSL1,HSD17B8,CBR4,ELOVL4,HACD4,ELOVL5,HACD2,EHHADH, ACADS,ACAT2,HADHB, ACADM,ELOV6
			MCAT, FASN,SCD5,FADS1,FADS2,ACADL,HSD17B4,HACD3
ko00564	Glycerophospholipid metabolism	4.12E-02	GNPAT, AGPAT5,ETNK2,PLPP1,DGKE, GPAT4,DGKB,GPD1,AGPAT1,PCYT1A,PLA2G15,PLD1,DGKA, PISD
			PLA2G4A,PLD3,LPGAT1,GPAM,PLA2G12A,DGKD, LPIN3,GPAT3
ko04911	Insulin secretion	4.25E-02	CAMK2G,ATP1A2,FXYD2,ADCY7,ADCY6,ADCYAP1R1,CREB5,KCNMB4,GCK,VAMP2,SLC2A2,CREB3L1
			CACNA1F,PRKACB, PRKCB,KCNN4,CAMK2D,STX1A,SLC2A1
ko04922	Glucagon signaling pathway	4.12E-02	SIRT1,PRKAA1,PPP3CB,SIK2,CAMK2G,PRKAG1,GCK,PPP3CC,SLC2A2,PRKACB, PHKG1,CALM3,SLC2A1
			PPARGC1A,CPT1B,CREB1,ITPR1,PKM,CALM1
ko04146	Peroxisome	3.51E-02	GNPAT, ACSL1,PEX1,PEX11A,IDH2,NUDT7,PEX19,PEX12,CROT,PEX6,DDO,ABCD1,FAR1,PEX7,DECR2
			PEX10,EHHADH,MVK,ABCD3, SLC25A17,ABCD4

In addition, several pairs of miRNA-mRNA targeting relationships of interest were identified, such as novel-m0035-5p—ACSL1, novel-m0035-5p—ELOVL4, bta-miR-677—ACSL1, bta-miR-485—FADS2, miR-129-x—ELOVL4, and miR-9-x—ACSL1. Studies have reported that variants of human fatty acid desaturase 1 (*FADS1*, encoding delta-5 desaturase) and fatty acid desaturase 2 (*FADS2*, encoding delta-6 desaturase) are associated with PUFAs (PUFA) in the blood and long-chain (LC) PUFA levels have a strong correlation (Glaser et al., 2010). *FADS2* gene polymorphism affects the content of unsaturated fatty acids (Gol et al., 2018; Proskura et al., 2019). In addition, studies have found that > C20 PUFAs synthesized by *FADS2* play an important role in regulating liver triacylglycerol and cholesterol accumulation during PUFA deficiency (Hayashi et al., 2021). As the key rate-limiting enzyme for the synthesis of >20 PUFAs, *FADS2* plays an important role in regulating the content of arachidonic acid (Stoffel et al., 2014). Hepatic steatosis was observed in *FADS2*-knockout mice that were fed a diet containing C18 PUFA but no C20 PUFA, and hepatic steatosis was reduced by administering arachidonic acid. Related studies have found that interference or overexpression of the *ACSL1* gene affects the expression of the downstream gene *COX2* and consequently the content of arachidonic acid (Cao et al., 2020).

In the study of *ACSL1* gene promoter transcription regulation, several important transcription factors, were identified among which *Sp1*, the main negative transcription regulator, significantly reduced the activity of the *ACSL1* promoter (Zhao et al., 2016). Interestingly, *Sp1* could bind to the promoter region of *FADS2* to increase the promoter activity of *FADS2* (Li et al., 2019). Thus, it was hypothesized that *ACSL1* acted as a regulatory switch between the *FADS2* and *COX2* genes, which worked together to maintain the homeostasis of arachidonic acid content in the body. Very long chain fatty acid extension-4 (ELOVL4) is a fatty acid condensing enzyme that mediates the biosynthesis of very long chain PUFAs (VLC-PUFA; ≥ C28) in a limited number of tissues (Agbaga et al., 2014). Previous studies have shown that *ELOVL4* is required for the synthesis of C28 and C30 saturated fatty acids (VLC-FA) and C28-C38 very long-chain PUFAs (VLC-PUFA) (Agbaga et al., 2008), which are present in retinal photoreceptors, skin, sperm, and testis, which play an important role in normal and long-term function (McMahon et al., 2011; Harkewicz et al., 2012). Similar to fatty acid synthesis, the activity of enzymes involved in fatty acid extension and desaturation appears to be regulated primarily at the transcriptional level rather than through post-translational protein modification (Guillou et al., 2010). Therefore, it is necessary to understand the regulation of *ELOVL4* by miRNAs as post-transcriptional regulators.

MicroRNAs bind to specific complementary sites of the target mRNA, thereby degrading mRNA or inhibiting its translation and regulating protein expression (Bartel, 2009). This study showed that novel-m0035-5p—ACSL1, novel-m0035-5p—ELOVL4, and bta-miR-485—FADS2 had the same differential trend. However, this did not imply that the key role of these miRNAs had been overlooked. The expression of miRNAs varied in different tissues and at different stages. In addition, miRNA coordinated biological functions by targeting genes. Therefore, the mutual targeting effects of different tissues and different stages were also different (Wienholds and Plasterk, 2005; Bushati and Cohen, 2007). In addition, *ACSL1*, *FADS2*, and *ELOVL4* were significantly enriched in fatty acid metabolism and fatty acid extension pathways. The three aspects of differential genes, signal pathways, and gene function were comprehensively considered to determine the miRNAs related to the synthesis of unsaturated fatty acids. Future studies would verify the functional regulation of unsaturated fatty acid synthesis by these miRNAs.

## Conclusion

RNA-Seq technology was used to identify miRNAs associated with UFAs synthesis by interfering with and none-interfering the *ACSL1* gene. Functional enrichment results indicated that DE-miRNAs were mainly involved in unsaturated fatty acid synthesis-related signaling pathways. Through the miRNA-mRNA interaction network, several key miRNA-mRNA targeting relationships were screened, including novel-m0035-5p—ACSL1, novel-m0035-5p—ELOVL4, miR-9-x—ACSL1, bta-miR-677—ACSL1, miR-129-x—ELOVL4 and bta-miR-485—FADS2. These miRNAs might regulate unsaturated fatty acid synthesis in bovine adipocytes by targeting these genes.

## Data Availability

The data presented in the study are deposited in the NCBI repository, https://www.ncbi.nlm.nih.gov/bioproject/PRJNA856126 and the accession number: PRJNA856126.

## Supplementary material

Supplementary File S1 Details of differentially expressed miRNAs.

Supplementary File S2 Details of differentially expressed miRNAs target genes.

Supplementary File S3 GO analysis of differentially expressed miRNAs target genes.

Supplementary File S4 KEGG analysis of differentially expressed miRNAs target genes.
